# Short pulse grid and subthreshold micropulse laser (the sandwich grid) plus intravitreal ranibizumab for the treatment of diabetic macular edema

**DOI:** 10.1186/s40942-024-00585-x

**Published:** 2024-09-30

**Authors:** Renato Peroni, José Augusto Cardillo, Rafael Memória, Tomas de Oliveira Castro Teixeira Pinto, Lucélia Albieri, Ingrid U. Scott, Rodrigo Jorge

**Affiliations:** 1 Division of Ophthalmology, Ribeirão Preto Medical School, University of São Paulo (USP), Ribeirão Preto, São Paulo, Brazil; 2 Departments of Ophthalmology and Public Health Sciences, Penn State College of Medicine, Hershey, PA USA

**Keywords:** Diabetic retinopathy, Diabetic macular edema, Subthreshold micropulse laser, Non-damaging retinal laser, Retinal photocoagulation, Retinal photostimulation, Intravitreal injection, Anti-VEGF, Ranibizumab

## Abstract

**Objective:**

To investigate the effects of two laser treatment procedures combined, short pulse grid laser (SP) and subthreshold micropulse laser (MP) (the sandwich grid [SWG] technique), plus intravitreal ranibizumab (IVR) on central subfield thickness (CSFT), best-corrected visual acuity (BCVA) and macular sensitivity in patients with diabetic macular edema (DME).

**Methods:**

Forty-five eyes (of 33 patients) with center-involving DME were treated with the SWG laser technique plus IVR and followed for 12 months. Laser treatment was performed at baseline: SP laser spots were placed in a grid pattern in the macular area (500 *µ*m from the fovea) according to the extension of DME; subsequently, MP laser was delivered up to the edge of the fovea. MP laser re-treatment sessions could be performed every 3 months if DME was present and CSFT was ≥ 300 μm on SD-OCT. IVR injection was performed at baseline and repeated monthly if CSFT > 300*µ*m. Preoperatively and monthly, ophthalmological examination was performed including measurements of BCVA, CSFT, and macular sensitivity.

**Results:**

One-year follow-up data is available for 37 eyes of 27 patients. Mean ± SE CSFT (µm) was 509.36 ± 25.14 and 325.76 ± 15.34 at baseline and 12 months, respectively. A significant reduction in mean CSFT was observed at all study visits compared to baseline (*p* < 0.001). Mean ± SE BCVA (logMAR) was 0.62 ± 0.04 and 0.45 ± 0.04 at baseline and 12 months, respectively. A significant improvement in mean BCVA was observed at all study visits compared to baseline (*p* < 0.001). Mean ± SE macular sensitivity (dB) was 17.85 ± 0.80 and improved to 19.05 ± 0.59 after one year of follow-up (*p* = 0.006). The mean number of IVR injections was 8.29 ± 0.63. The mean number of MP laser procedures including the initial SWG laser session was 3.67 ± 0.22. No ocular or systemic adverse effects were observed.

**Conclusion:**

The SWG laser technique plus IVR was associated with significant improvement in macular edema, BCVA, and macular sensitivity in patients with center-involving DME.

**Clinical Trial Number (CAAE):**

22969019.4.0000.5440.

## Introduction

Diabetic retinopathy is the leading cause of blindness among working-age adults globally [1]. Vision loss related to diabetic retinopathy may compromise independence and impair quality of life [2]. The most common cause of visual loss in diabetic patients is diabetic macular edema (DME) [3] In 1985, the Early Treatment Diabetic Retinopathy Study (ETDRS) demonstrated the beneficial effects of macular argon laser photocoagulation in the management of clinically significant macular edema; laser treatment was associated with a 50% reduction in the risk of moderate visual loss (loss of ≥ 3 lines) by 3 years [4]. Subsequentially, laser photocoagulation emerged as the mainstay treatment for DME. Laser treatment, however, damages retinal tissue and is associated with a risk of such adverse effects as enlargement of the laser scars, scotomas, choroidal neovascularization, and potential loss of central vision [5, 6]. According to a recent Diabetic Retinopathy Clinical Research Network (DRCR.net) [7], the modified Early Treatment Diabetic Retinopathy Study (mETDRS) [8] technique (visible endpoint) is the approach used most commonly by ophthalmologists in the United States for DME laser therapy.

Enhanced comprehension of laser interactions and the retinal healing response, as well as a desire to prevent tissue injury and associated adverse effects, have led to new laser therapy modalities and strategies [9–19]. Subthreshold diode micropulse laser (SDML) was developed as a laser treatment alternative to the Early Treatment Diabetic Retinopathy Study (ETDRS) suprathreshold laser photocoagulation and its originated variants. SDML therapy delivers repetitive very short “ON time” laser pulses separated by relatively long “OFF time” intervals within a single exposure envelope [20, 21]. This advance allows the surgeon to achieve an efficacious subvisible (non-visible) endpoint and selective laser application confined to the retinal pigment epithelium (RPE), reducing thermal elevation and heat conduction through adjacent targeted tissue, limiting laser-induced retinal damage, and optimizing the therapeutic effect [9, 19, 21, 22] Short pulse duration grid laser (SP) consists of a shorter exposure (10 to 20ms) continuous wave pulse, leading to a lighter and smaller visible endpoint tissue photocoagulation, and a localized thermal effect with minimal heat spread [23–25]. Intravitreal anti-VEGF therapy is the current preferred treatment for center-involving DME [26–28], and its significant anatomic and functional effects have been well-demonstrated in randomized clinical trials [29–31]. The purpose of this current prospective study is to investigate the effects of two laser treatment procedures combined, short pulse grid laser (SP) and subthreshold micropulse laser (MP) (the sandwich grid [SWG] technique), plus intravitreal ranibizumab (IVR) on central subfield thickness (CSFT), best-corrected visual acuity (BCVA) and macular sensitivity in patients with diabetic macular edema (DME).

## Methods

The study adhered to the tenets of the Declaration of Helsinki and was approved by the local research ethics committee of the School of Medicine of Ribeirão Preto at the University of Sao Paulo. All patients evaluated in the Retina Section of the Department of Ophthalmology, School of Medicine of Ribeirao Preto of the University of Sao Paulo with DME in at least 1 eye were invited to participate in the study, and written informed consent was obtained before study entry.

### Study population

Inclusion criteria were as follows: (1) patients *≥* 18 years of age; (2) center-involving DME with CSFT ≥ 300 μm on spectral-domain optical coherence tomography (SD-OCT) (Spectralis, Heidelberg, Germany); (3) ETDRS BCVA between 0.3 logMAR (Snellen equivalent: 20/40) and 1.6 logMAR (Snellen equivalent: 20/800); (4) no prior macular laser therapy for DME. Exclusion criteria were as follows: (1) vitreomacular traction or epiretinal membrane on SD-OCT; (2) history of intraocular surgery within the last 6 months, except those who underwent cataract surgery; (3) retinal photocoagulation outside macular area (including pan-retinal photocoagulation) within 4 months; (4) proliferative diabetic retinopathy needing pan-retinal photocoagulation (PRP); (5) macular capillary dropout on fluorescein angiography; (6) history of glaucoma or ocular hypertension (defined as an intraocular pressure > 22 mmHg); (7) an ocular condition (other than diabetic retinopathy) that, in the opinion of the investigator, might affect macular edema or alter visual acuity during the course of the study; (8) systemic corticosteroid therapy; (9) had undergone intraocular antiangiogenic therapy within 2 months; (10) previous thromboembolic advent including cerebrovascular accident and acute myocardial infarction within 6 months; (11) other clinical trial participation in the previous 30 days. The demographic characteristics of the study population are presented in Table 1.

**Table 1:** Baseline characteristics of patients treated with short pulse Grid and Subthreshold Micropulse Laser (the Sandwich Grid) PLUS Intravitreal Ranibizumab for the Treatment of Diabetic Macular Edema

Baseline Characteristics	Descriptive Measures
Sex (male/female)	19/28
Age (years) (mean ± SE)	61 ± 8.21
LogMAR BCVA (mean ± SE)	0.61 ± 0.04
Central subfield thickness (mean ± SE)	509 ± 25.14
Macular sensitivity (mean ± SE)	17.89 ± 0.79
Duration of diabetes (years) (mean ± SE)	15.4 ± 7
HbA1c (mean ± SE)	8.52 ± 0.35

### Outcomes

The primary outcome measure was the mean change in CSFT. Secondary outcomes were mean change in BCVA and mean change in macular sensitivity threshold.

### Treatment procedures

Patients underwent detailed ophthalmologic evaluation at baseline (week 0) and every 4 weeks up to week 52. Baseline examination included measurement of BCVA according to the standardized ETDRS refraction protocol, applanation tonometry, slit-lamp biomicroscopic examination, indirect fundus examination, fundus color photography, fluorescein angiography (FA) (HRA; Heidelberg Engineering, Heidelberg, Germany), microperimetry (MP) (MAIA microperimeter; CenterVue, Padova, Italy) using a Goldmann III stimulus size covering a 10° diameter area with 37 measurement points for macular sensitivity assessment, and SD-OCT evaluation (Spectralis Eyetracker Tomographer, HRA-OCT; Heidelberg Engineering) including CSFT measurements. Examination was repeated at 4-week follow-up visits except for fundus color photography, FA, and MP, which were performed at baseline and weeks 16, 28, 40 and 52. The hemoglobin A1c (HbA1c) test was collected at baseline and weeks 28 and 52.

### Intravitreal injections

All injections were performed using topical proparacaine drops under sterile conditions. Before injections, the eyelids were scrubbed with 10% povidone-iodine, and 5% povidone-iodine drops were applied to the conjunctiva directly over the intended injection site [32]. Ranibizumab (0.5 mg/ 0.05 cc; Novartis Pharma Stein AG, Stein, Switzerland) was injected into the vitreous cavity using a 29-gauge 0.5-inch needle inserted through the inferotemporal pars plana 3.0–3.5 mm posterior to the limbus. Subsequently, central retinal artery perfusion was confirmed with indirect ophthalmoscopy. Patients were instructed to instill 1 drop of 0.3% ciprofloxacin into the injected eye 4 times daily for 1 week after the procedure. Intravitreal ranibizumab injection retreatment was performed monthly if central subfield macular thickness was greater than 300 μm.

### Laser treatment

**Fig. 1:**
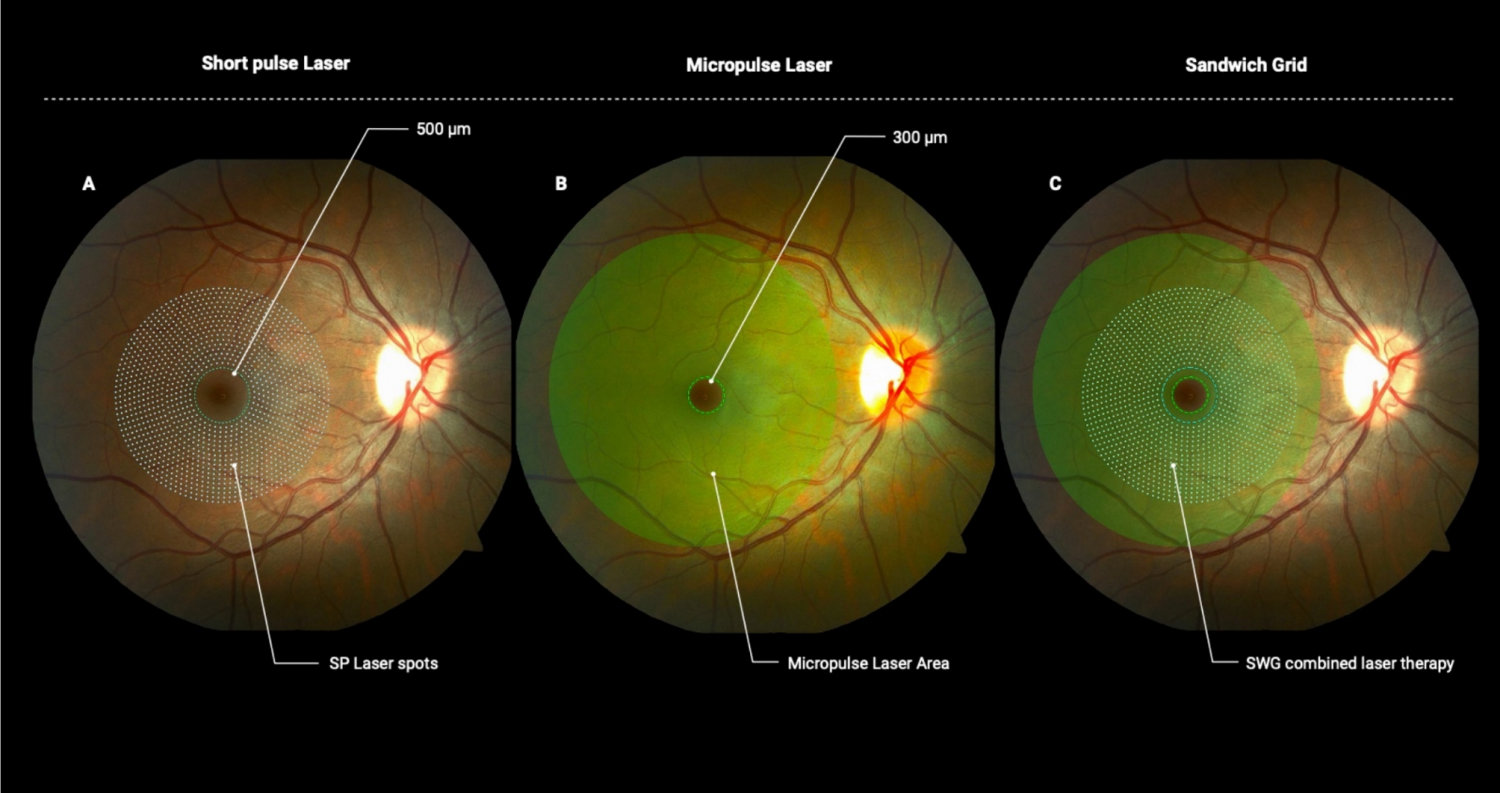
Schematic representation of sandwich grid (SWG) laser approach. Illustration depicting the shortpulse laser treatment area, maintaining a 500 μm distance from the foveal center, with one spot spacing between marks **(A)**. Micropulse laser treatment area with confluent invisible spots covering the entire macula up to a 300 μm distance from the foveal center (B). Illustrative scheme of combined SWG laser therapy applied in an overlapping manner: SP and SDML **(C)**

Macular laser treatment was performed after pupillary dilation and topical anesthesia. The short pulse treatment protocol was performed with a continuous wave 532-nm green laser (Purepoint Laser; Alcon, Fort Worth, TX) with the following parameters: 100 μm spot size, 20 ms duration, power set to a barely visible burn, number of spots varied according to the area of DME, one burn width apart in a grid pattern performed up to 500 μm from the center of the foveal avascular zone. Focal coagulation of microaneurysms was not considered. Short pulse photocoagulation was delivered once during the baseline visit. Micropulse diode laser treatment was performed subsequentially at the same visit with an 810 nm diode laser (Fastpulse Laser; Opto, São Carlos, SP, Brazil) with the following parameters: 125 μm spot size, 15% duty cycle of 200 ms. Power was set individually for each patient from an initial continuous wave test burn titrated upward until a white burn was produced in a location outside the macular area. Micropulse mode was set by increasing the power obtained in the test burn by 20%. Spots were delivered continuously confluent in the entire macula up to 300 μm from the center of the foveal avascular zone as shown in Fig. 1. Micropulse treatment could be repeated every 3 months if DME was present and CSFT was ≥ 300 μm on SD-OCT.

### Statistics

Statistical analyses were performed using IBM SPSS Statistics for Windows, version 23.0 (IBM Corporation, Armonk, NY, USA), and included data frequency and descriptive measures such as mean, standard deviation, standard error, minimum and maximum. For analysis of continuous variables (visual acuity, CSFT, and macular sensitivity), the Kolmogorov-Smirnov test was first applied, to investigate which variables followed a normal distribution, and it was determined that only the variable macular sensitivity followed a normal distribution; thus, macular sensitivity was analyzed using ANOVA and the Student t-test for paired samples. As for the visual acuity and CSFT variables, we performed the Mann-Whitney tests for comparisons of means and the Wilcoxon paired samples test. Finally, to test the correlation between the continuous variables investigated, we used the Spearman correlation. For categorical variables (visits, laser sessions, and ranibizumab injections), we used the chi-square test to investigate the association between variables. All statistical analyses were considered significant when the p-value < 0.05.

## Results

Twenty-seven patients (37 eyes) completed 1 year of follow-up. The participants had a mean age of 61 years (SE ± 8.21). The average baseline HbA1c was 8.52%. All participants had a history of type 2 diabetes mellitus.

**Fig. 2:**
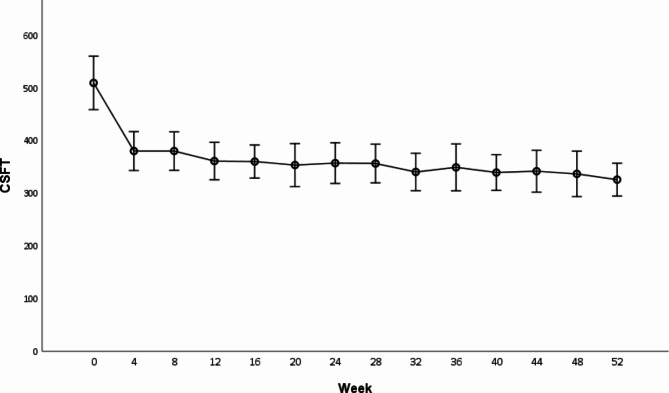
Mean CSFT (µm) ± SEM at the 14 study visits (baseline to week 52). There was significant improvement in CSFT at each study visit compared to baseline (* = *p* < 0.001)

At baseline, the mean CSFT was 509.36 ± 25.14 μm. A significant reduction to 325.76 ± 15.34 μm was observed at one-year follow-up (*p* < 0.001), representing a mean reduction of 183.6 μm compared to baseline. A significant decrease in CSFT compared to baseline was observed at all follow-up visits (*p* < 0.001), with the maximum reduction observed at week 52 (Fig. 2).

**Fig. 3:**
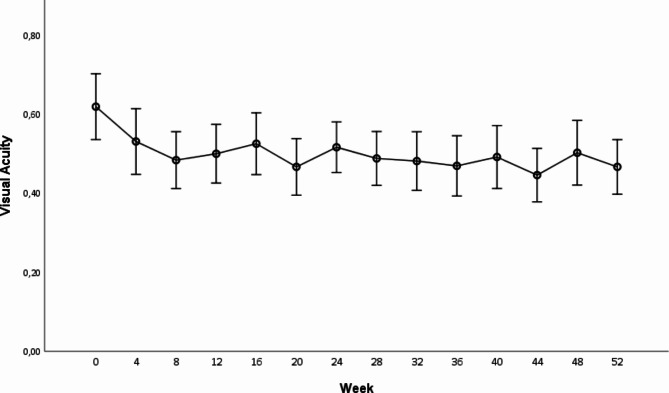
Mean Best-corrected Visual Acuity (LogMAR) ± SEM changes across the 14 study visits (baseline to week 52). There was significant improvement in BCVA at each study visit compared to baseline (* = *p* < 0.05)

Mean baseline BCVA (logMAR) ± standard error (SE) was 0.62 ± 0.04 (Snellen equivalent of approximately 20/80). At the one-year follow-up, the mean logMAR BCVA had improved to 0.45 ± 0.03 (*p* < 0.001), with a mean improvement compared to baseline of 0.17 logMAR (this corresponds to an improvement of approximately 1.7 ETDRS lines or 8.5 ETDRS letters). The maximum improvements in BCVA were observed at weeks 44 and 52 (Fig. 3).

**Fig. 4:**
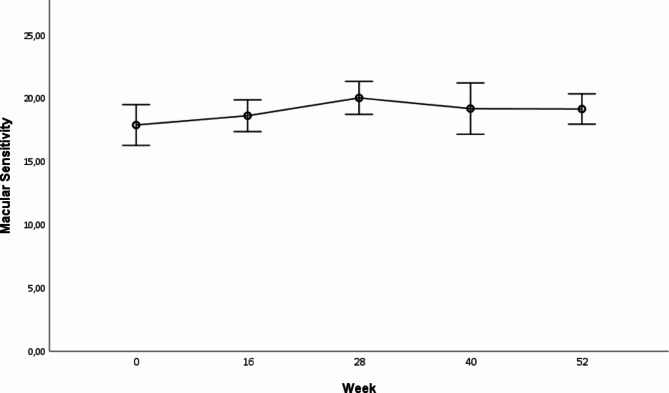
Mean macular sensitivity (dB) ± SEM at the 14 study visits (baseline to week 52). There was significant improvement in macular sensitivity by the end of the study period compared to baseline (* = *p* < 0.05)

Baseline mean macular sensitivity threshold ± standard error (dB), as assessed with microperimetry, was 17.85 ± 0.80 dB and improved to 19.05 ± 0.59 dB at week 52, with a mean sensitivity gain of 1.2 dB compared to baseline (*p* = 0.006) (Fig. 4). Notably, analyses revealed significant associations between BCVA and CSFT as well as between BCVA and macular sensitivity, with p-values less than 0.001 for both correlations.

**Fig. 5:**
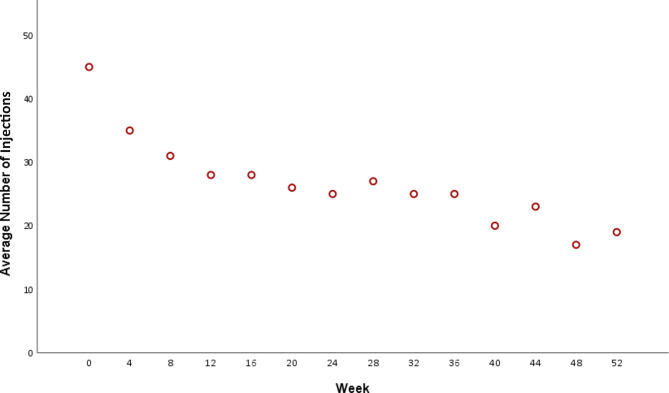
Plot summarizing the distribution of the average number of intravitreal (IV) injections of ranibizumab for the management of diabetic macular edema up to week 52

The mean number of intravitreal ranibizumab injections administered was 8.29 ± 0.63, (maximum possible injections, 14) (Fig. 5) and the mean number of laser sessions as per research protocol (based on CSFT) was 3.67 ± 0.22 including first visit baseline treatment. For SP and SDML laser treatments, the mean number ± SD of laser spots delivered in all sessions were 283.5 ± 55 and 716.45 ± 36.19, respectively. The mean power levels (mW) ± SD were 131.33 ± 20.29 and 298.72 ± 41.03 for the SP and SDML treatments, respectively.

**Fig. 6:**
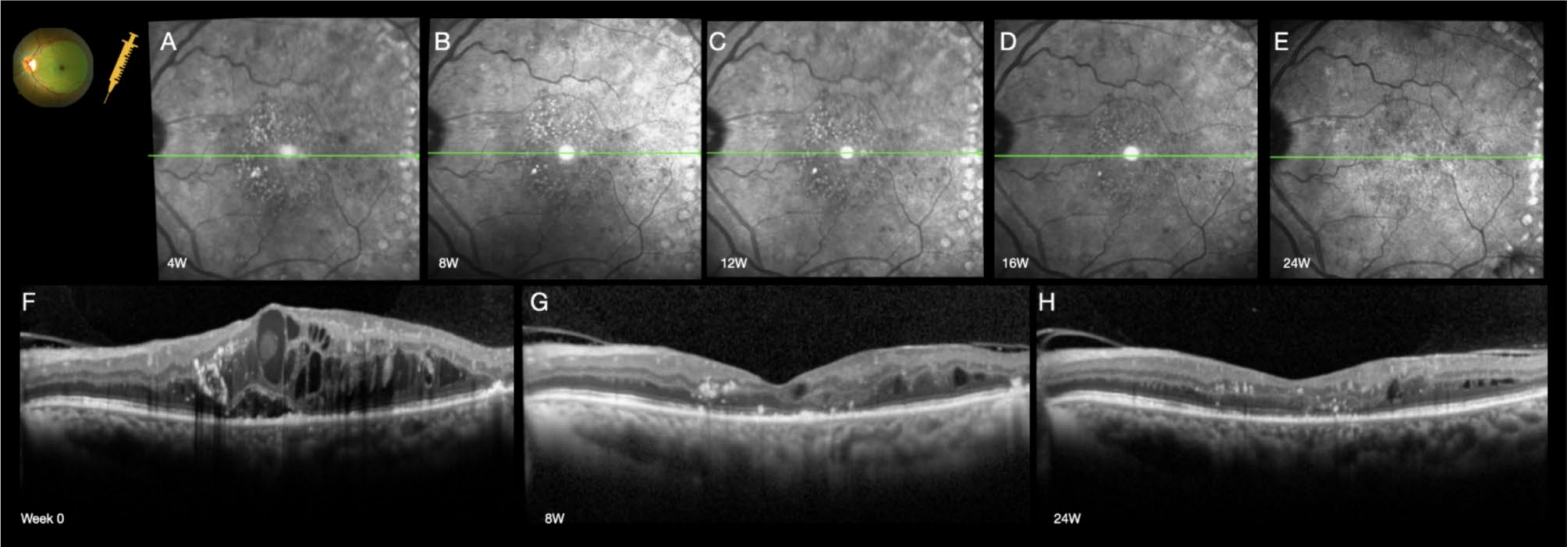
Treatment of diabetic macular edema (DME) with combined laser treatment (Sandwich grid) and intravitreal Ranibizumab performed at the initial visit following established parameters, with no additional treatment sessions up to the 24-week follow-up. A total of 289 short pulse laser spots and 693 micropulse laser shots were applied. Only one intravitreal injection of ranibizumab was needed. Near-infrared imaging illustrating hyperreflective dots within the perifoveal area (SP laser spots), with progressive reduction of hyperreflectivity over time **(A-E)**. SD-OCT illustrates extensive DME with intraretinal and subretinal fluid, along with a baseline visual acuity of 20/160 (Snellen equivalent) **(F)**, followed by significant and sustained anatomical and functional improvement to 20/50 at week 8 **(G)**, and further progress to 20/32 at week 24 **(H)**

Intravitreal injections were performed by retinal surgeons and all laser sessions were performed by one of the authors (RP). No ocular or systemic adverse effects were observed in any of the patients. As expected, mild laser burns were observed in patients after short pulse grid laser photocoagulation (SP), and no additional visible laser burns were observed in eyes after subthreshold micropulse laser (SDML) as observed in Fig. 6.

## Discussion

This study describes a new strategy in laser therapy for the treatment of DME, combining two macular laser techniques: short pulse (SP) as a shorter exposure macular laser photocoagulation and subthreshold diode micropulse laser (SDML) described as macular laser photostimulation [33]. Based on a Medline search, this is the first study to combine these laser techniques with anti-VEGF therapy for DME treatment.

Macular laser photocoagulation is generally performed delivering high energy to retinal tissue with resultant laser-induced retinal damage, which is not fundamental to obtain a therapeutic effect [33]. Very white laser burns are implicated in the formation of retinal scars, retinal atrophy, and subsequent functional decline. For this reason, we investigated laser techniques that do not leave any laser burn (SDML) or that leave barely visible laser burns (SP).

SDML and SP employ lower laser energy, aiming for precision and sparing of the neurosensory retina. The specific mechanism of action for SDML remains elusive, despite several existing hypotheses. SDML stimulation results in well-confined temperature increases that are sublethal to retinal cells and stimulate retinal pigment epithelium (RPE) cells. SP laser photocoagulation uses selective and shorter exposure pulses (20ms) targeting the photoreceptor outer segments and RPE, leading to the restoration of the RPE barrier after laser injury [34–36]. Their therapeutic responses combined may be attributed to such factors as regulation of inflammatory cytokine expression, reduction of VEGF, upregulation of heat shock proteins, modulation of gene expression, alterations in Muller cells’ metabolic activity, and other potential mechanisms, either individually or in conjunction [18, 37–42]. 

In our study, we observed a substantial reduction in CSFT, with a mean baseline CSFT of 509.36 μm and an improvement of 183.6 μm. This compares favorably with pivotal studies in the field. For instance, the RESTORE study, which evaluated ranibizumab monotherapy, ranibizumab combined with laser treatment, and laser monotherapy for DME, reported mean CSFT reductions of 128.3 μm and 118.7 μm in the ranibizumab plus laser group and the ranibizumab alone group, respectively [43] Similarly, the DRCR.net Protocol I study assessed the efficacy of intravitreal ranibizumab with either prompt or deferred laser therapy, and reported significant reductions in mean CSFT from a baseline of 371 μm and 382 μm in the respective groups to 131 μm and 137 μm, respectively, after one year [44] Although the reduction in CSFT in our study aligns with these trials, it is noteworthy that our study population experienced a CSFT reduction of larger magnitude. This may be attributable, at least in part, to the fact that our study population had a higher CSFT at baseline and, therefore, had room for more CSFT improvement.

Our study population also demonstrated a significant improvement in mean BCVA of 0.17 logMAR (8.5 letters) from the baseline visit to the end of the one-year follow-up. This improvement in BCVA is correlated with the observed decrease in CSFT. In comparison, larger trials using laser and anti-VEGF for DME treatment reported a better baseline mean BCVA, like the DRCR.net Protocol I [44] in which the baseline mean BCVA was20/63, compared to our study’s baseline of approximately 20/80 Snellen equivalent. Nonetheless, the improvement in BCVA over 12 months in our study aligns with findings from these larger trials. This is exemplified by the RESTORE study [43], which documented a mean 6.4-letter gain, situating our outcomes within the spectrum observed in comparable research [29, 45, 46].

While micropulse laser photostimulation is not expected to cause retinal damage, short pulse photocoagulation may transiently affect macular sensitivity during the outer retinal restructuring process [34] even with the lower laser exposure required in this protocol. For this reason, during the one-year follow-up period, microperimetry tests were conducted to assess the impact of DME on retinal sensitivity. We observed a mean macular sensitivity gain of 1.2 dB at week 52 compared to at baseline (*p* = 0.006). This is in contrast to traditional focal laser photocoagulation, which is associated with decreased retinal sensitivity in macular sectors post-photocoagulation for DME, accompanied by a reduction in outer nuclear layer thickness [42]. 

In our study, the average number of intravitreal ranibizumab injections administered over one year was 8.29, which aligns with the findings of major studies like the RESTORE [43] and Protocol T [45] studies, in which the mean numbers of anti-VEGF injections were 7 and 10, respectively. However, studies exploring combination treatments, such as the one by Moisseiev et al., demonstrated that combined micropulse laser and anti-VEGF therapy required fewer intravitreal injections compared to anti-VEGF monotherapy (1.7 vs. 5.6) [47] Other similar research also found that combination treatments were associated with fewer anti-VEGF injections while achieving comparable BCVA outcomes [48, 49]. This difference may be related to the quality of glycemic control. Notably, the higher average mean HbA1c level in our study (8.52%) compared to that in the DRCR Protocol T ranibizumab group (7.8%) and in the RISE/RIDE ranibizumab groups (7.7%/7.6%), suggests a potential disparity in diabetes management among the patients in the various trials. This could be indicative of poorer diabetes control in our study population, possibly due to socioeconomic factors and healthcare access. These baseline characteristics are generally associated with worse DME control with anti-VEGF treatment [50–52].

In addition to the development of promising pharmacological agents for the treatment of DME in recent years, developments in laser phototherapy employing personalized parameters may also contribute to optimal anatomical and functional outcomes, with the potential to reduce the number of anti-VEGF injections required which, in turn, would lower treatment costs and injection-related adverse events. While the present study provides evidence that the SWG laser technique plus IVR is effective in reducing CSFT and improving BCVA and macular sensitivity in patients with center-involving DME, the lack of a control group treated with IVR only precludes definitive conclusions to be drawn with respect to the potential independent adjunctive effect of SWG laser. In addition, our study included a small sample size and there is a learning curve of the proposed laser technique, which may influence the reproducibility of this technique for specialists unfamiliar with SDML and SP laser therapy.

## Data Availability

No datasets were generated or analysed during the current study.
